# Age-related subproteomic analysis of mouse liver and kidney peroxisomes

**DOI:** 10.1186/1477-5956-5-19

**Published:** 2007-11-27

**Authors:** Jia Mi, Itsaso Garcia-Arcos, Ruben Alvarez, Susana Cristobal

**Affiliations:** 1 Department of Cell and Molecular Biology, Biomedical Center, Box 596, Uppsala University, SE-751 24 Uppsala, Sweden; 2 Department of Biochemistry and Biophysics, The Arrhenius Laboratories for Natural Sciences, Stockholm University, SE-106 91 Stockholm, Sweden

## Abstract

**Background:**

Despite major recent advances in the understanding of peroxisomal functions and how peroxisomes arise, only scant information is available regarding this organelle in cellular aging. The aim of this study was to characterize the changes in the protein expression profile of aged versus young liver and kidney peroxisome-enriched fractions from mouse and to suggest possible mechanisms underlying peroxisomal aging. Peroxisome-enriched fractions from 10 weeks, 18 months and 24 months C57bl/6J mice were analyzed by quantitative proteomics.

**Results:**

Peroxisomal proteins were enriched by differential and density gradient centrifugation and proteins were separated by two-dimensional electrophoresis (2-DE), quantified and identified by mass spectrometry (MS). In total, sixty-five proteins were identified in both tissues. Among them, 14 proteins were differentially expressed in liver and 21 proteins in kidney. The eight proteins differentially expressed in both tissues were involved in β-oxidation, α-oxidation, isoprenoid biosynthesis, amino acid metabolism, and stress response. Quantitative proteomics, clustering methods, and prediction of transcription factors, all indicated that there is a decline in protein expression at 18 months and a recovery at 24 months.

**Conclusion:**

These results indicate that some peroxisomal proteins show a tissue-specific functional response to aging. This response is probably dependent on their differential regeneration capacity. The differentially expressed proteins could lead several cellular effects: such as alteration of fatty acid metabolism that could alert membrane protein functions, increase of the oxidative stress and contribute to decline in bile salt synthesis. The ability to detect age-related variations in the peroxisomal proteome can help in the search for reliable and valid aging biomarkers.

## Background

Aging is a natural phenomenon that affects the entire physiology of an organism. It is a complex process resulting from changes in the expression and regulations of numerous genes over time. It has become evident from high-throughput studies that the metabolic pathways affected in aging are interconnected [1,2]. Therefore, techniques such as proteomics that allow the simultaneous analysis of thousands of molecular parameters within a single experiment could facilitate to identify candidate proteins for aging biomarkers in animal models.

Comparative proteomics has been used to study the effect of aging on the proteome from rat skeletal muscle [3], in epithelial cells [4], brain mice [5,6], and on specific organelles such as Golgi apparatus, and endoplasmic reticulum [7] or mitochondrial proteins in mice [8], in rat [9], in bovine heart [10] or rat brain [11]. These techniques have been applied to examine the effect of anti-aging agents on human endothelial cells [12]. Comparative studies using premature aging Hutchinson-Gilford progeria syndrome fibroblasts revealed differential protein expression and glycosylation of membrane proteins [13]. Proteomics studies on aged samples have also disclosed various non-enzymatic modifications such as glycosylation and nitration that progress with age [14]. These studies clearly indicate the value of additional comprehensive proteomic analysis to find novel aging biomarkers.

It is widely accepted that the reactive oxygen species (ROS) are one of the mediators of aging in most species [15], either being a direct cause of aging or as a by-product of a genetically programmed process [16]. So far, most of the subcellular studies have been focused on the mitochondrion that generates the main proportion of cellular ROS. Likewise, the peroxisomal oxidative metabolism is an additional source of ROS. The peroxisome also responds to oxidative stress and protects against oxidative damage. However, the information about which process initiates the aging cascade in the peroxisome is still scarce.

Studies have reported a general decrease in peroxisomal function with aging [17]. Decreases in catalase (CAT) activity has been found in various studies [17,18]. But in particular, inconsistencies have been reported about the age-related effects in the peroxisomal β-oxidation [19]. Recently, peroxisome senescence has been studied in human fibroblasts showing that aging comprises the peroxisomal targeting signal protein import and the key antioxidant enzyme CAT [20]. The lack of peroxisomal CAT in the nematode *Caenorhabditis elegans *has been shown to cause a progeric phenotype [21].

Our recent improvements in a comparative proteomic technique aimed for concentrating the subproteome of interest avoiding a complex cellular fractionation [22]. We have recently characterized peroxisome-enriched fractions from two mouse tissues: liver and kidney by 2-DE based proteomics [23], predicted peroxisomal proteomes from sequenced genomes [24], and analyzed peroxisomal proteomes from invertebrate species [25]. Those studies on invertebrates provided the largest number of identified proteins from mussel's peroxisomal proteome and the proteins have been utilized to identify novel and poor understood pathways affected by xenobiotics [22,26,27].

Applying comparative proteomics to peroxisomal samples could provide new clues to which molecular events were associated with aging in peroxisomes. In this paper, peroxisome-enriched fractions from two mouse tissues: liver and kidney and three ages: 10 weeks, 18 months, 24 months were analyzed by quantitative proteomics. First, we present tissue-specific protein expression profiles from the different ages and a common protein profile to both tissues; thereafter, differentially expressed proteins were identified by MALDI-TOF MS and the differentially expressed proteins were functionally classified. Finally, Western blotting and analysis of predicted transcription factors are in agreement with the quantitative proteomic data. Our results provide an age-related subproteomic analysis of a mouse peroxisome-enriched fraction from liver and kidney, two tissues with different generation capacity.

## Results

### Quantitative analysis of the peroxisome-enriched fraction from young and old mouse liver and kidney

**Figure 1:**
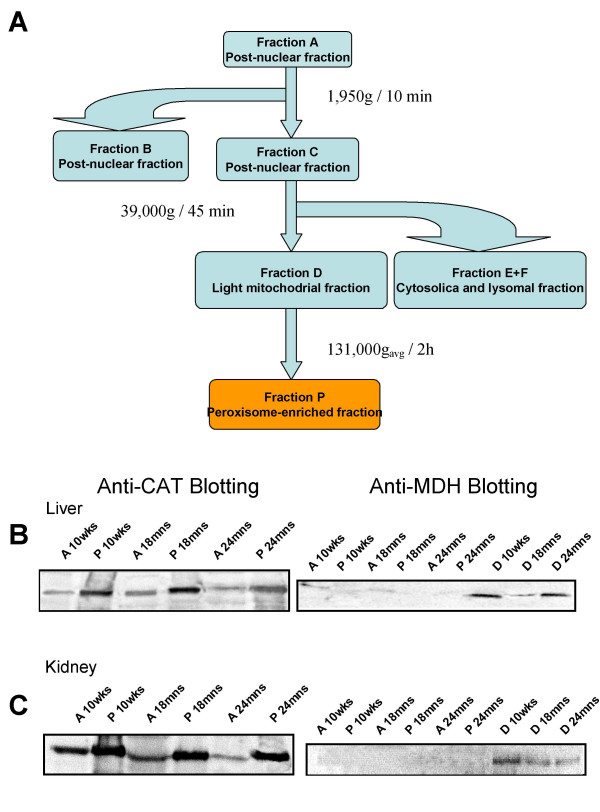
Scheme of the protein purification process in **A **and immunoblot analysis of the peroxisome-enriched fractions in **B **and **C. **The enrichment of specific organelle proteins was followed by loading the same amount of protein (20 μg each) of total homogenate (lane A), light mitochondrial fraction (laneD) and highly purified peroxisomal fraction (lane P) onto a 12.5% T polyacrylamide gel. In **B **section, the immunoblot of CAT were more intensively stained in the fraction P than A and in **C **section, MDH immunoreaction is found mainly in fraction D. CAT, catalase; MDH, malate dehygrogenase.

The principal aim of this study was to characterize age-dependent changes in the peroxisomal proteome of liver and kidney. The tissues were subjected to differential centrifugation and the peroxisome-enriched fraction was further purified with an iodixanol gradient in a density gradient centrifugation (Fig. 1A). The quality of each isolation procedure was assessed. The purity of the peroxisome-enriched fraction was based on the measurement of the marker enzyme, CAT, and verified by protein gel blot analysis routinely [28]. The quality of each isolation procedure was assessed and the possible cross-contamination with other organelles was followed by protein gel blot analysis (Fig. 1B and 1C). The method provided preparations from different tissues and ages with equivalent quality [23]. Proteins were subjected to 2-DE followed by colloidal Coomassie staining. Statistical analyses were applied to compare the average spot ratio of expression between the 2-DE maps from tissues of different ages. On average, about 140 spots were identified in both tissues that corresponded to 65 different proteins [see Additional file 1] [22].

**Figure 2:**
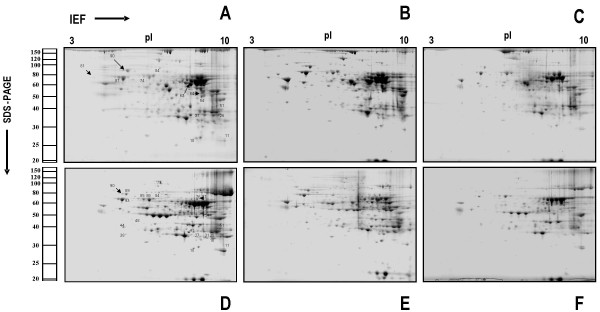
Representatives 2-DE gels from peroxisome-enriched fractions were isolated from liver and kidney of *M. musculus *and separated by 2-DE with denaturing isoelectric focusing (IEF) on immobilized pH gradients in the first dimension between pH 3–10 non-linear (11 cm) and SDS-PAGE 12.5% in the second dimension. Gels were calibrated for molecular mass (in kDa) and pI (in pH units) by external pH and mass standards and stained by colloidal Coomassie. Differential expressed proteins are marked with numbers in A for liver and D for kidney. **A. **tissue: liver, age: 10 weeks; **B. **tissue: liver, age: 18 months; **C. **tissue: liver, age: 24 months; **D. **tissue: kidney, age: 10 weeks; **E. **tissue: kidney, age: 18 months; **F. **tissue: kidney, age: 24 months.

**Figure 3:**
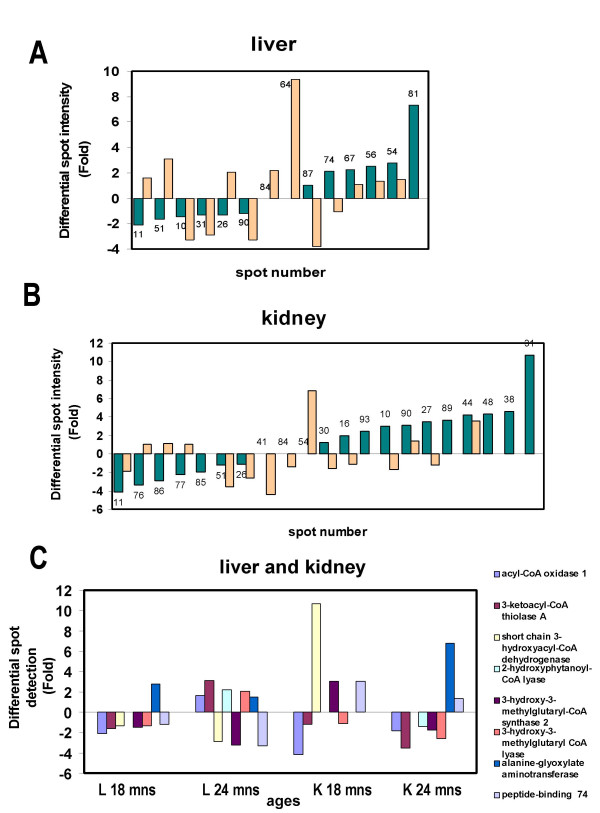
Proteins differentially expressed in the 18 months and 24 months versus 10-weeks old group. The ratios were calculated dividing the volume percentage per each spot from the 18 months or 24 months by the volume percentage per spot in the 10 weeks old group. The vertical axis corresponds to the average ratio of expression, above the 0 value for the up-regulated proteins and below the 0 value for the down-regulated ones. According to 18 months old group, in the horizontal axis the down-regulated proteins are organized with the lowest values on the left side and the up-regulated ones show the highest values on the right side. Color code: green for 18 months group and orange for the 24 months old group. **A. **Liver age-related proteins. **B. **Kidney age-related proteins. **C. **Differential spot detection in fold from the 8 proteins composing the common age-related PES to both tissues. The four groups correspond to 18 months liver, 24 months liver, 18 months kidney and 24 months kidney samples.

In the liver, 14 spots showing changes in protein expression between young and old mouse tissues was observed. The 18 months samples showed a down-regulation response that was compensated by an up-regulation at 24-month. The differentially expressed proteins are illustrated in Fig. 2A, B and 2C, Fig. 3A and Table 1. In the kidney, the statistically significant difference in protein expression affected to 21 spots. The variations at 18 months were equally distributed between up- and down-regulations whereas at 24 months, down-regulation was the main aging-associated effect. The differentially expressed proteins in the kidney are illustrated in Fig. 2D, E and 2F, Fig 3B and Table 2. Eight proteins were up or down-regulated under all condition studied. These proteins composed an aging protein expression profile common to liver and kidney. The most of these eight proteins were down-regulated in the 18 months liver and 24 months kidney samples. However, in the 24 months old liver samples, these proteins were mainly up-regulated (Fig 3C).

**Table 1:** Subset of age-associated proteins from quantitative proteomic analysis of liver peroxisomal proteins. Proteins were identified by MALDI-TOF MS ^a)^.

**10 wks**		**18 mns**		**24 mns**									
**A (Fold)**	**SDV(Fold)**	**B (Fold)**	**SDV(Fold)**	**C (Fold)**	**SDV(Fold)**	**Pathway**	**Location**	**NCBI nr**	**Cvage**	**Score**	**p**	**Mr ob**	**pI ob**
**1**	**0.29**	**-2.12**	**0.03**	**1.63**	**0.22**	**β-oxidation**	**pero**	gi|66793429	24%	60	0.1	25000	9
**1**	**0.31**	**-1.62**	**0.47**	**3.11**	**0.52**	**β-oxidation**	**pero**	giI30525893	26%	66	0.0251189	40000	9.0
**1**	**0.48**	**0.00**	**0.00**	**2.20**	**0.52**	**α-oxidation**	**pero**	gi|31560355	30%	104	3.981E-06	65000	6.8
**1**	**0.15**	**-1.48**	**0.34**	**-3.28**	**0.06**	**isoprenoid biosynthesis**	**pero**	gi|20965433	33%	68	0.0158489	26000	8.7
**1**	**0.50**	**-1.31**	**0.39**	**2.05**	**0.69**	**isoprenoid biosynthesis related**	**pero**	gi|409499	24%	70	0.01	33000	9.1
**1**	**0.42**	**2.78**	**0.43**	**1.48**	**0.23**	**amino acid metabolism**	**pero**	gi|19388006	24%	63	0.0501187	42000	7.8
**1**	**0.09**	**1.02**	**0.25**	**-3.77**	**0.13**	**putative peroxisomal prot**	**pero**	gi|6678726	18%	60	0.1	67000	6.1
**1**	**0.43**	**2.14**	**0.39**	**-1.09**	**0.41**	**putative peroxisomal prot**	**pero**	gi|6753036	40%	117	1.995E-07	60000	6.6
**1**	**0.18**	**-1.33**	**0.10**	**-2.87**	**0.13**	**β-oxidation**	**mito**	gi|21431780	24%	55	0.3162278	33000	8.8
**1**	**0.21**	**0.00**	**0.00**	**9.36**	**0.15**	**β-oxidation**	**mito**	gi|20810027	23%	70	0.01	46000	7.8
**1**	**0.14**	**2.26**	**0.62**	**1.09**	**0.29**	**glutamate biosynthesis**	**mito**	gi|6680027	22%	64	0.0398107	60000	7.4–7.5
**1**	**0.38**	**-1.20**	**0.15**	**-3.31**	**0.21**	**stress response**	**mito**	gi|14917005	25%	63	0.0501187	75000	6.2
**1**	**0.32**	**2.52**	**0.53**	**1.32**	**0.38**	**glutamine metabolism**	**cyt**	gi|15419027	28%	59	0.1258925	42000	7.5
**1**	**0.05**	**7.35**	**0.46**	**0.00**	**0.00**	**protein folding**	**cyt**	gi|129729	28%	64	0.0398107	61000	5

^a) ^Protein numbers correspond to number from the 2-DE gels shown in Figure 2. Data from molecular mass in kDa and pI can assist the localization in the Figure 2. The 10 weeks old group correspond to A, 18 months old group as B and 24 months old group as C. The 10 weeks old group has been taken as a reference. Therefore, the change in protein expression associated with age is presented as the ratio A/A or B/A or C/A for liver. The ratios were calculated dividing the volume percentage per each spot in the 10 weeks, 18 months or 24 months divide by the volume percentage per spot in the 10 weeks. The standard deviations data are included. The biochemical pathways and subcellular localization are also indicated.

**Table 2:** Subset of age-associated proteins from quantitative proteomic analysis of kidney peroxisomal proteins. Proteins were identified by MALDI-TOF MS ^a)^.

**10 wks**		**18 mns**		**24 mns**									
**D (Fold)**	**SDV(Fold)**	**E (Fold)**	**SDV(Fold)**	**F (Fold)**	**SDV(Fold)**	**Pathway**	**Location**	**NCBI nr**	**Cvage**	**Score**	**p**	**Mr ob**	**pI ob**
**1**	**0.22**	**-3.4**	**0.04**	**1.02**	**0.06**	**auxiliary β-oxidation**	**pero**	gi|6753272	30%	114	3.981E-07	60000	7.5–7.8
**1**	**0.18**	**-2.27**	**0.02**	**1.04**	**0.12**	**auxiliary β-oxidation**	**pero**	gi|6753272	30%	114	3.981E-07	60000	7.5–7.8
**1**	**0.31**	**-4.16**	**0.07**	**-1.86**	**0.10**	**β-oxidation**	**pero**	gi|66793429	24%	60	0.1	25000	9
**1**	**0.16**	**-1.18**	**0.46**	**-3.53**	**0.13**	**β-oxidation**	**pero**	giI30525893	26%	66	0.0251189	40000	9.0
**1**	**0.26**	**0**	**0.00**	**-4.38**	**0.35**	**α-oxidation**	**pero**	gi|6754564	20%	64	0.0398107	36000	7.3
**1**	**0.12**	**0**	**0.00**	**-1.44**	**0.22**	**α-oxidation**	**pero**	gi|31560355	30%	104	3.981E-06	65000	6.8
**1**	**0.29**	**3.04**	**0.58**	**-1.73**	**0.05**	**isoprenoid biosynthesis**	**pero**	gi|20965433	33%	68	0.0158489	26000	8.7
**1**	**0.18**	**-1.11**	**0.22**	**-2.61**	**0.15**	**isoprenoid biosynthesis related**	**pero**	gi|409499	24%	70	0.01	33000	9.1
**1**	**0.14**	**0**	**0.00**	**6.80**	**1.10**	**amino acid metabolism**	**pero**	gi|19388006	24%	63	0.0501187	42000	7.8
**1**	**0.17**	**4.6**	**0.36**	**0.00**	**0.00**	**amino acid metobolism**	**pero**	giI17390882	25%	65	0.0316228	33000	6.0
**1**	**0.10**	**-1.95**	**0.16**	**0.00**	**0.00**	**amino acid metobolism**	**pero**	gi|477004	32%	104	3.981E-06	65000	6.5
**1**	**0.27**	**-2.88**	**0.14**	**1.14**	**0.19**	**amino acid metobolism**	**pero**	gi|477004	32%	104	3.981E-06	65000	6.7
**1**	**0.23**	**1.24**	**0.05**	**-1.59**	**0.15**	**purine/pyrimidine metabolism**	**pero**	gi|18044669	26%	63	0.0501187	35000	9.0–9.1
**1**	**0.27**	**3.45**	**0.52**	**-1.21**	**0.13**	**putative peroxisomal protein**	**pero/cyt**	gi|226778	24%	67	0.0199526	33000	7.5
**1**	**0.19**	**2.46**	**0.45**	**0.00**	**0.00**	**putative peroxisomal protein**	**pero**	gi|76779273	21%	59	0.1258925	62000	5.7–5.9
**1**	**0.14**	**1.94**	**0.22**	**-1.17**	**0.37**	**β-oxidation**	**cyt**	gi|13182962	39%	60	0.1	30000	9.0
**1**	**0.14**	**10.66**	**0.10**	**0.00**	**0.00**	**β-oxidation**	**mito**	gi|21431780	24%	55	0.3162278	33000	8.8
**1**	**0.18**	**4.3**	**0.17**	**0.00**	**0.00**	**β-oxidation**	**mito**	gi|26345684	25%	67	0.0199526	45000	6.5
**1**	**0.19**	**4.26**	**0.62**	**3.58**	**0.63**	**glycolysis**	**mito**	gi|18043470	24%	54	0.3981072	37000	6.1
**1**	**0.24**	**3.61**	**0.71**	**0.00**	**0.00**	**stress response**	**mito**	gi|14917005	25%	63	0.0501187	75000	6.0
**1**	**0.20**	**3.06**	**0.22**	**1.37**	**0.43**	**stress response**	**mito**	gi|14917005	25%	63	0.0501187	75000	6.2

^a) ^Protein numbers correspond to number from the 2-DE gels shown in Figure 2. Data from molecular mass in kDa and pI can assist the localization in the Figure 2. The 10 weeks old group correspond to D, 18 months old group as E and 24 months old group as F. The 10 weeks old group has been taken as a reference. Therefore, the change in protein expression associated with age is presented as the ratio D/D, E/D and F/D for kidney. The ratios were calculated dividing the volume percentage per each spot in the 10 weeks, 18 months or 24 months divide by the volume percentage per spot in the 10 weeks. The standard deviations data are included. The biochemical pathways and subcellular localization are also indicated.

### Principal component analysis (PCA) and hierarchical clustering

**Figure 4:**
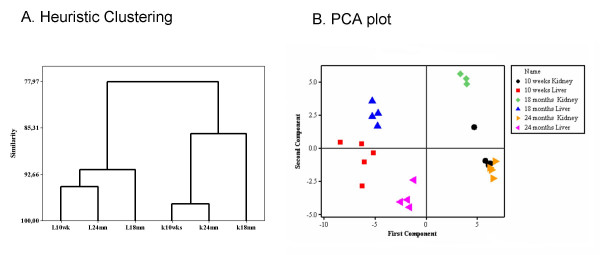
Organization of data by multivariate analyses. **A. **Heuristic clustering plot **B. **PCA performed on correlation matrix.

Cluster analysis methods were applied to confirm the age-related changes observed in the peroxisome-enriched fractions. Hierarchical clustering was used to blindly classify similar gels into classes. All liver gels were clustered together with a 93% similarity level and the kidney gels showed up to 84% similarity. It is remarkable that independently from the tissue, the similarity between the 10 weeks and 24 months gels were close to 95% and were clearly separated from the 18 months gels. For the kidney samples, the clustering differences between the controls and the 24 months samples was stronger with 13.3% difference than the 2% difference between the control and the 18 months samples (Fig 4A).

PCA clearly distinguished 2-DE gels based on tissues and ages (Fig 4B). The first component provided a sharp separation between tissues. In the positive side of the x-axis were situated all kidney gels and in the negative side, the corresponding liver gels. The second component separated the different ages. In the positive side of the y-axis the 18 months old gels from both tissues were situated and on the opposite side, the 24 months old gels also from liver and kidney. The control gels from young tissues were situated in the boundary between positive and negative values in the second component.

### Cross-validation of some MALDI-TOF MS identified proteins by immunochemical analysis

**Figure 5:**
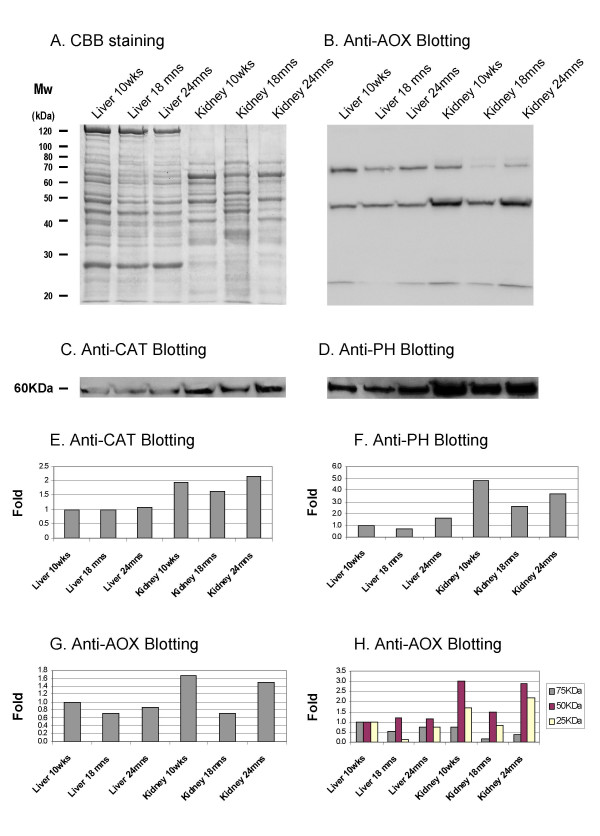
SDS-PAGE, immunoblotting from the different peroxisome-enriched fractions. **A. **The increase in protein expression in a one-dimensional gel was followed by loading the same amount of protein (20 μg each) of the peroxisome-enriched fraction from liver and kidney samples of 10 weeks old, 18 months old and 24 months (lane P) onto a 12.5% T polyacrylamide gel and stained by Coomassie blue. **B. **Immunoblot against AOX antibody.**C. **Fragment from the immunoblot against CAT antibody. **C. **Fragment from the immunoblot against PH antibody. **E. **Plot of the differential intensities from the immunoblot against anti-CAT, **F. **anti-PH and **G. **anti-AOX for the total and H. Values from the three subunits that immunoreact. In the x-axis, different group organized by tissue and age are represented and in the y-axis, differential band intensity in fold. The value from the band of the immunoblot was normalized against the 10 weeks old liver sample.

Approximately 140 spots were tryptic-digested and analysed by MALDI-TOF MS and proteins were identified by combined data obtained from servers available for the academic communities [24,29-33]. Here, we confirmed the identification of 65 proteins from the liver and kidney peroxisome-enriched factions [23]. In addition, the protein identification by peptide mass fingerprinting was cross-validated with immunochemical analysis for several proteins (CAT, acyl-CoA oxidase (AOX) and multifunctional protein) (Fig. 5A, B C and 5D). The Western blot data from AOX, CAT and multifunctional protein showed firstly, a recovery of these enzymes in the 24 months versus the 18 months group and secondary, a tissue-specify response. However, the different sensitivity range of these two techniques, 2DE-based analysis and Western blot analysis does recommend a qualitative comparison rather than a quantitative data comparison.

### Functional classification of the differentially expressed proteins and prediction of transcriptional factors

**Figure 6:**
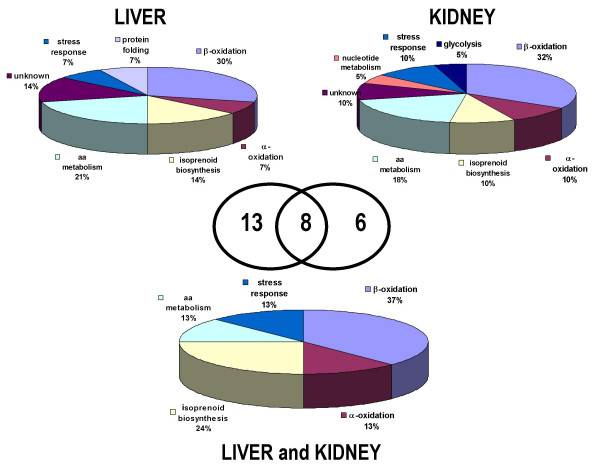
Functional classification of the age-related changes in the peroxisomal proteome from liver and kidney. Distribution of protein profiles according to biochemical pathways in a pie-representation: in liver, kidney and common to both tissues. In the center, a Venn diagram of the number of proteins that composes the aging protein profile for each tissues and the common to both of them.

Twenty-seven proteins showed significant age-dependent differences in protein expression. These proteins were classified in Table 1 and 2 by subcellular localization and functional pathways. In the subcellular classification, the majority of these differentially expressed proteins were peroxisomal proteins, around 20% were mitochondrial proteins and 14% corresponded to cytosolic proteins (Fig. 6). Proteins were divided into 9 main biochemical pathways: β-oxidation, α-oxidation, isoprenoid biosynthesis, amino acid metabolism, purine and pyrimidine metabolism, stress response, protein folding and glycolysis and protein with unknown function. The β-oxidation was the pathway more affected by the effect of age in both tissues.

Each cell type or tissue at specific age or developmental stage has its own characteristic gene expression profile that could be defined, to some extent, by the presence of a combination of transcription factors. In order to find additional evidences to the age-related changes in the peroxisomal proteome of liver and kidney, we searched for predicted transcription factors in each of the 65 identified genes. The transcription factors were classified by different factors such as score, e-value, conservation among different genomes and predicted models [see Additional file 2]. In Table 3, we presented transcription factors from *Mus musculus *that were present in genes from the differentially expressed proteins. None of the factors were found in all eight genes from the common expression profile. Studying which transcription factors appeared in those genes, the R-ALPHA and SPZ1 transcription factors were found in 5 out of those 8 genes. Six different transcription factors appeared in proteins from the β-oxidation pathway and five in the pathways of the isoprenoid biosynthesis. From the 50 predicted transcription factors included in the list, 6 of them were conserved among different genomes whereas most of them were specific from *M. musculus*.

**Table 3:** Predicted transcription factors from the genes of the most relevant age-related proteins in this study.

	**β-oxidation**				**α-oxidation**	**isoprenoid bio.**		**aa met.**		**nucl.met.**		
**TF vs PROT**	**CAT**	**ACOX1**	***ACCA1A***	***EHHADH***	***PHYH***	**HMGCS2**	**HMGCL**	**AGXT**	**DAO1**	**MRGPRF**	**Conserved**	**Model**
**RXR-ALPHA**	1	1	1	1	1	1	1				no	T01331
**SPZ1**	1	1	1	1	1		1	1	1	1	yes	MA0111
**NF-Y**	1	1	1	1			1				yes	M00775
**SP-1**	1	1		1	1	1		1		1	no	M00931
**PAX**	1	1			1	1	1	1	1	1	no	M00979
**PPARG**	1	1				1		1			no	M00512
**ZIC**	1	1							1		no	M00448
**BSAP**	1			1				1			no	MA0014
**COEI**	1			1							no	T01112
**CAC-BINDING PROTEIN**	1			1							no	M00720
**HOX-1.3**	1					1				1	yes	M00023
**KROX**	1					1				1	no	M00982

**SOX**		1	1	1		1		1	1		no	MA0078
**MEF-1,2**		1	1					1	1		no	T00505
**BRACHYURY**		1	1					1			no	MA0009
**MTF-1**		1		1					1		no	T00515
**ZF5**		1		1							no	M00333
**ARNT**		1			1		1		1	1	no	MA0004
**IRF-1**		1			1			1	1		no	M00062
**EGR**		1			1			1	1		no	M00807
**PPAR DIRECT REPEAT 1**		1				1					no	M00763
**PPAR , HNF-4, COUP, RAR**		1				1					no	M00762
**N-MYC**		1					1				no	MA0104
**ELF1**		1							1		no	M00746

**PPARALPHA:RXR-ALPHA**			1	1		1			1		no	M00518
**C_EBP**			1	1		1					no	M00912
**NF-KAPPAB**			1	1							no	M00774
**CRX**			1		1	1		1			no	M00623
**C/EBPBETA**			1		1	1					no	M00109
**LYF-1**			1			1		1	1		no	T00479
**FOX**			1			1		1	1		no	T04203
**HSF**			1				1	1	1	1	no	T00384

**CP2**				1		1					yes	M00072
**SF-1**				1		1					no	T01147
**EVI-1**				1			1			1	no	M00081
**AHR-ARNT**				1			1		1		no	MA0006
**CART-1**				1			1				yes	T03999
**ALX-4**				1			1				yes	T02967

**CREB**					1					1	no	M00114

**SRF**						1	1	1	1		no	M00922
**MYOD**						1	1		1	1	no	M00929
**MYOGENIN **						1	1		1	1	no	M00712
**NKX2-2**						1		1			no	T02384

**E2A**							1		1	1	no	M00804
**USF**							1			1	no	M00796

**MAF**								1	1		no	M00983
**PU.1**								1		1	no	T00702

**LMO2 COMPLEX**									1	1	no	M00277
**P53 DECAMER**									1	1	no	M00761
**TCF-1(P)**									1	1	no	M00670

## Discussion

Cell aging is a multifactorial process: DNA damaged and repaired, telomeres shortened, aberrantly posttranslational modified proteins, alteration in protein expression, and cellular damage by accumulation of ROS are some of the factors that contribute to the general decline in physiological functions [1]. Studying aging at the organelle level has been attracting attention mainly in mitochondria, where the free radical theory of aging was focused. However, information is scarce in other organelles such as peroxisomes [23]. The peroxisome together with the mitochondrion are the main cellular ROS producers [34] therefore, a quantitative proteomic analysis of peroxisomal samples could provide some functional classification of the differentially expressed proteins in aging.

The aim of this study was to identify differential expressed proteins in liver and kidney from young versus old mice. We have recently addressed that quantitative proteomics could address the tissue-specific variation of the peroxisomal proteome [23]. Therefore, using a subproteomic technique improved in our laboratory [23,25], we could show that the age-related peroxisomal response was tissue-specific. In the liver, few proteins with moderate variations composed the age-related protein expression signature (PES) whereas in kidney, the age-related PES was formed by a higher number of proteins with stronger variations. The Western blot analyzes with antibodies against CAT, AOX or the multifunctional protein also showed a tissue-specify response. In the case of oxidative stress, which play a fundamental role in the aging process in peroxisomes [35], differential expressed proteins associated with stress response were solely observed in kidney but not in liver. In other peroxisomal studies using the nematode *Caenorhabditis elegans*, it has been reported that genes that shorten life-span include a variety of stress response genes, among them genes encoding catalases [21]. Age-associated changes of CAT and antioxidant enzymes have been also described in organs of rats [36].

Data from statistical analysis also confirmed the tissue-specific difference in the peroxisomal proteome with aging. Using PCA, liver and kidney samples were clearly separated by the first component. From the protein classification in biochemical pathways, we found a liver-specific biochemical pathway, protein folding and a liver-specific biochemical pathway, nucleotide metabolism and pathways that were differentially expressed in both tissues such as the isoprenoid biosynthesis pathway. Similarly in agreement with what our data showed, Lee *et al*. [37], also reported age-related tissue-specific response between three different tissues: brain and gastrocnemius and muscle. In that study, brain and gastrocnemius showed similar up-regulation of stress response but differed from the muscle response to aging.

The tissue-specific response to aging could be discussed in the context of the liver and kidney different regeneration capacity. Liver has an impressive restorative capability and it is the only organ in the body that is capable of regenerating itself after damage whereas, the kidney cortex is used as an organ with a restricted regeneration capability [38]. It is remarkable that aging did not impair the liver regeneration capacity [39]. It has been suggested that the liver high regeneration capacity could be correlated to the constant level of telomerase activity through out life in contrast to kidney, where only traces of telomerase activity can be detected after few weeks [40]. In liver, differentiated cells proliferate without dedifferentiation in a tightly controlled process involving inflammatory cells growth factors, and hormones [41]. However in the kidney, the majority of the cells that divide to repair the injured tubules comes from an endogenous cell population rather than from bone marrow-derived cells [42].

Only eight proteins were found to be common to the age-related PES from kidney, and liver. The two of these proteins from the β-oxidation pathway, AOX, and 3-ketoacyl-CoA thiolase A were down-regulated in the 18 months old samples. In liver, the diminution of activity on the β-oxidation pathway has been reported in association with aging at different levels. On one hand, changes in the membrane fatty acid composition could arise from reducing the degradation of saturated and monounsaturated very long fatty acid. These changes could be associated with alteration in membrane protein function, insertion, and signal transduction [43]. On the other hand, the less efficient peroxisomal β-oxidation would lead to the accumulation of very long chain fatty acids in the tissues. This accumulation is known to be toxic for the organism and may induce symptoms similar to those from genetic peroxisomal disorders [44].

Proteins from the isoprenoid biosynthesis pathway also composed this common PES. The role of peroxisomes in the biosynthesis of cholesterol has been under discussion during the last years. Several studies have indicated that the early steps in the cholesterol biosynthesis could occur in peroxisomes [45,46]. However, the subcellular localization of the enzymes mevalonate kinase, phosphomevalonate kinase, and mevalonate pyrophosphate decarboxylase has been questioned [47-49]. Recent publications clearly showed that the first part of the cholesterol synthesis from acetyl-CoA to farnesildiphosphate occurs in peroxisomes and refute the hypothesis that peroxisomes were not involve in the biosynthesis of isoprenoids [50,51]. Aging compromises many hepatic functions, among them, the age-related decline in bile salt secretion could be caused by the decline in bile salt synthesis. In particular, the decrease in the peroxisomal rate-limitating enzyme of the biosynthesis of cholesterol, HMG-CoA synthase, in the aged samples could partially explain the decline of bile salt secretion. It has been speculated that HMG-CoA lyase and HMG-CoA reductase could compete for the peroxisomal HMG-CoA [51,52]. The presence of HMG-CoA lyase in peroxisomes has been studied with different experimental approaches [53-55] and in a recently published paper is also included in the isoprenoid biosynthesis pathway [51]. In liver, we observed that the expression of both proteins decreased in the 18 months group. However, in the oldest group, the decrease of the HMG-CoA reductase and the increase of the enzyme HMG-CoA lyase could cooperate reducing the HMG-CoA availability for the cholesterol biosynthetic pathway.

Another protein from this common PES was the 2-hydroxyphytanoyl-CoA lyase which is localized in peroxisomes and dependent on thiamine pyrophosphate and Mg^2+ ^[55]. This is a low expressed protein in kidney [23] therefore, the peroxisomal α-oxidation was not particularly studied in this tissue. Deficiencies in this essential cofactor in the mammalian metabolism of 3-methyl-branched fatty acids could be related to a reduction of the enzymatic activity. In humans, it has been reported that the lower thiamine pyrophosphate concentrations in elderly people was related to age itself than to co-existent illnesses [56]. Finally, one mitochondrial protein, short chain 3-hydroxyacyl-CoA dehydrogenase (SCHAD), participates in this aging signature. The high levels of the SCHAD in the 18 months kidney were remarkable high. The SCHAD is important in brain development and aging. It has been reported that abnormal levels of this enzyme in brain may contribute to the pathogenesis of some neural disorders and aging. This protein has been considered as a potential target for intervention in Alzheimer's and Parkinson's diseases [57], as this is one of the enzymes that have affinity for amyloid β-peptide [58]. It is likely that the elevated level of this protein is a factor in this pathogenesis. The mitochondrial SCHAD seems to play also an important role in brain development and aging, even though glucose and not fatty acids is the major energy source of the nervous system. However, the SCHAD role in aging in other tissues has not been reported yet.

## Conclusion

In summary, we have isolated peroxisome-enriched fractions from two mouse tissues: liver and kidney, at three different ages: 10 weeks, 18 months, 24 months. The peroxisomal proteomes were analyzed by quantitative proteomics. First, we showed an age-related PES that was common to both tissues and a tissue-specific PES. Secondly, these findings at the protein level could be interpreted in combination with the transcription factors prediction data. Our results could indicate some age-related peroxisomal dysfunctions including: the alteration the fatty acid metabolism that could alter membrane protein functions; the decrease of CAT in kidney that may contribute to oxidative stress and the upregulation of isoprenoid biosynthesis that could contribute to decline in bile salt synthesis. This is the first age-related proteomic analysis of the peroxisomal matrix in two tissues with different regeneration capacity. Our results indicate that quantitative subproteomic approaches can provide some insights into possible mechanism that control organelle aging and can be of help in the search for reliable and valid aging biomarkers.

## Methods

### Animals

Male C57bl/6J mice from 10 weeks old were obtained from B & K Universal AB (Sollentuna, Sweden). Male C57bl/6J mice from 18 months old and 24 months old were obtained from Janvier laboratories (Le Genest-St-Isle, France). Young and old animals were feed with equivalent type of diet by their respective laboratories. Old animals were carefully transported to Sweden by plane and maintained at Uppsala BMC animal house for acclimatizing. Ten animals were utilized in each of the experimental group. All animals from the three groups were kept under normal animal house conditions with food and water *ad libitum*. Mice were fasted overnight and euthanized by CO_2 _treatment followed by cervical dislocation. Livers and kidneys were excised, dried with filter paper and weighed. They were immediately minced in ice-cold homogenization buffer (HB): 250 mM sucrose, 5 mM Mops, 1 mM EDTA, 0.1% ethanol, 2 mM PMSF, 1 mM DTT, 1 mM ε-aminocaproic acid, 2 μM leupetin, 2 μM pepstatin.

### Subfractionation of intact peroxisomes

Homogenization of the minced tissue in pools and subcellular fractionation by differential centrifugation were performed according to an established method [59] with a few modifications outlined below. The main subcellular fractions were termed according to the nomenclature used by Völk and Fahimi [60]. Thus, the total homogenate was termed A, the heavy mitochondrial fraction B, the light mitochondrial or peroxisome-enriched fraction D, the cytosolic fraction E and the microsomal fraction F. Two ml of D fraction was carefully layered on top of 15 ml of 28% iodixanol (v/v), 5 mM MOPS, 0.1% ethanol, 1 mM tetrasodium EDTA solution (pH 7.3, density 1.15) and 2 ml of 50% iodixanol (v/v) cushion (density 1.26) and centrifuged at 40 000 rpm (131 000 g_avg_) for 2 h in a Beckman L7-55 centrifuge using a TFT50.2 Ti rotor. The peroxisomal enriched fraction was obtained from the interface between 28% and 50% of iodixanol. The activities of following marker enzymes were measured in all the fractions: catalase (CAT) for peroxisomes, succinate dehydrogenase for mitochondria and acidic phosphatase for lysosomes [28]. Protein was determined according to Bradford [61] and Smith [62]. To analyze the quality of the peroxisomal fractions, we conducted Western blot analysis with different commercial polyclonal antisera, according to standard procedures, using chemoluminescence for detection [59].

The quality of each isolation procedure was assessed. The purity of the peroxisome-enriched fraction was based on the measurement of the marker enzyme, CAT and verified by protein gel blot analysis routinely [28]. Cross-contamination with other organelles was followed by protein gel blot analysis using antibodies against the mitochondrial protein malate dehydrogenase and by enzymatic activity assays of non-peroxisomal proteins: succinate dehydrogenase as mitochondrial marker of and acidic phosphatase for the lysosomes. In the different tissues and experimental groups, the peroxisome-enriched fraction quality was assessed and the criteria applied was to compare only fractions with enrichment of CAT activity between 14–16 fold and less than 1% of cross-contamination were applied to 2-DE.

### Protein extraction

The proteins were extracted by trichloroacetic acid/acetone precipitation. First, equal volume of 20% trichloroacetic acid in acetone containing 0.07% β-mercaptoethanol was added to the peroxisome-enriched fraction and the sample was kept at -20°C to reach the complete precipitation. The sample was centrifuged at 125 000 g for 15 min at +4°C. The supernatant was discarded and the precipitate was washed twice with 1 ml acetone containing 0.07% β-mercaptoethanol. The precipitate was dried for 30 minutes at room temperature.

### 2-DE PAGE

The 2-DE PAGE procedure was described in Mi *et al*. [26] with some small modifications. The samples were solubilized in a buffer A containing 7 M urea, 2 M thiourea, 2% CHAPS (w/v), 65 mM DTT, 2% Pharmalyte pH 3–10 (GE healthcare), bromophenol blue and rehydration solution B composed by 8 M urea, 2% CHAPS (w/v), 15 mM DTT, 1% β-mercaptoethanol (v/v), 0.2% pharmalyte pH 3–10. The gels for quantitative analysis contained 300 μg of proteins. The samples were applied onto immobilized pH 3–10 non-linear drystrips (11 cm for comparison gels, Bio-Rad). Isoelectric focusing was performed on a Protean IEF Cell (Bio-Rad) at 20°C with the following program: passive rehydration for 12 h, 250 V for 15 min, 8000 V for 2 h, reached 35000 Vh finally. The focused immobilized pH gradient strips were reduced (2% DTT) and alkylated (4% iodoacetamide) in equilibration buffer (6 M urea, 50 mM Tris-HCl, pH 6.8, 30% glycerol, 2% SDS). The second dimension was performed with Criterion pre-cast gels system (Bio-Rad). The pre-cast gels (12.5% acrylamide, Tris-HCl gels, 13 cm × 8.3 cm, 1 mm thick) were performed in Criterion dodeca cell (12 gels) at 120 V until the blue line reached the bottom. The pI and Mr scales of the 2-DE maps were internally calibrated by mixing molecular markers (Sigma) with samples before 2-DE analysis. For external calibrations, molecular mass markers (Bio-Rad) were loaded onto the second dimension. The protein spots were visualized by staining with Coomassie Brilliant Blue R 250 [63].

### Image capture and analysis

After staining, all gels were scanned with an image scanner II (GE healthcare, Uppsala), and the data were analyzed with a standard analysis process including spots detection, quantification and normalization, data analysis and statistics using ImageMaster™ 2D Platinum version 6.01 software from GE healthcare (Uppsala, Sweden). For the standardization, all gel images sensitivity was controlled for the detection of approximately 200 spots. Two match sets were constructed for liver and kidney peroxisome-enriched fractions. For each match set, three submatchsets were built based on ages. There were at least 4 replicates in each sub-matchset. The gels with most matched spots were defined as match-masters. The match-masters from different organs and ages were matched to each other. To accurately compare the measurements of spots in different gels, the normalized volume for a spot is calculated by dividing its volume by the total volume of the detected spots on the image. Normalized volumes from different spots on sample from kidney gels were compared against the corresponding spots on liver gels. The changing ratios and mean relative difference in spot intensity were calculated and obtained from the comparison window of the software.

### Protein identification by MALDI-TOF MS

Coomassie Brilliant Blue R 250-stained protein spots were excised from the gel and prepared for mass spectrometry (MS). The peptide extract (1 μL) from each tryptic digest using Ziptip C18 (Milipore) was crystallized in 0.5 μL of matrix solution (α-cyano-4-hydrocynnamic acid in methanol; Hewlett-Packard, Böblingen, Germany) on the matrix-assisted laser desorption/ionization-time of flight (MALDI-TOF) target plate (Applied Biosystems Inc). A MALDI-TOF MS, equipped with a nitrogen laser and operating in reflector/delay extraction mode (Voyager-DE-STR; Applied Biosystems Inc.) was used to obtain molecular mass information of the peptides. All MALDI-TOF spectra were internally calibrated using trypsin autodigestion peptides (842.51 Da and 2211.11 Da). The external calibration was done with Sequazyme Peptide Mass Standards Kit (Applied Biosystems). The raw data was refined by the software Data Explorer (Applied Biosystems) including baseline correction, noise filter, peak de-isotoping. This analysis was performed for both kidney and liver 2-DE maps.

### Database searching and analysis

The refined peaks from Data Explorer was submitted to online Server Mascot [30] and to proteinprospector [29] to match known proteins or translated open reading frames in databases at the National Center for Biotechnology Information (NCBI) and SWISS-Prot. The taxonomy was limited to *Mus musculus *and the peptide tolerant was set as 50 ppm, up to 1 missed cleavage was allowed. The protein identification was confirmed if its searching score was more than the cut-off and the estimated molecular weight corresponded to the theoretical Mw (Table 1 and 2). The identified proteins and the subcellular localization were confirmed by information in databases and predictions. Predictions for peroxisomal localization were made using the software programs PSORT [32], PTS1 predictor [33] and PeroxiP [24], for functional domains, Pfam [31], and [64].

### Cluster analysis methods

Using the Image master Platinum 6.0 and MINITAB 14 statistical software, the data was processed using two different kinds of multivariate analysis. PCA is a useful tool for data categorization, since it separates the dominating features in the dataset. Initially, PCA was performed, including proteins present in at least 80% of the 2-DE maps and after gap-ratio filtering (gap>1). Secondary, a hierarchical clustering was performed using the same spot selection criteria.

### Protein identification by immunoblotting

We conducted a protein gel blot analysis with different antibodies both, commercially available polyclonal antisera (Rockland) and provided from other researchers, according to standard procedures, using chemoluminescence for detection. For immunolocalization, polyclonal monospecific antisera for immunolocalization against rat liver AOX and PH were kindly provided by Prof, H. Dariush Fahimi (Heidelberg University, Germany) and the CAT antibody was commercially available. The specificity of those antisera against mussels peroxisomal proteins has been previously established by immunoblotting and immunoelectron microscopy [26,65].

### Prediction of transcription factors

A list of genes that correspond to the *M. musculus *peroxisomal proteome was created from National Center of Biotechnology Information [66] and Ensembl [67]. A list of predicted transcription factors, models and hits were generated using the ID gene as input data in with the program Mapper [68]. The selection of the transcription factors with high probability was based on the highest score (probabilistic measure of the match between the hit and model) values and the lowest E-values (measure of the likelihood of the hit being retrieved by chance). It was highlighted those sites with evolutionarily conserved regions. Tables with complete data can be found in supplementary material. Finally, a list of transcription factor was generated that were common to several biochemical pathways with information about E- value and the score.

## Abbreviations

2-DE, two- dimensional electrophoresis; MS, mass spectrometry; ROS, reactive oxygen species; CAT, catalase; PCA, principal component analysis; AOX, acyl-CoA oxidase; PES, protein expression signature; HMG-CoA, 3-hydroxy-3-methylglutaryl-CoA; SCHAD, short chain 3-hydroxyacyl-CoA dehydrogenase.

## Authors' contributions

Jia Mi: AB, JY, and ES.

Itsaso Garcia-Arcos: AB

Ruben Alvarez: MT

Susana Cristobal: FG and ES.

All authors read and approved the final manuscript.

## Supplementary Material

Additional file 1 Identification of proteins from mouse peroxisomal enriched fractions. Proteins were identified by MALDI-TOF-MS. Click here for file

Additional file 2 Predicted transcription factors from the genes of the identified proteins in this study. Click here for file
