# Supplementary material for: Age-related subproteomic analysis of mouse liver and kidney peroxisomes
Source: Proteome Sci. 2007 Nov 27;5:19. doi: 10.1186/1477-5956-5-19 (PMC2231346; doi:10.1186/1477-5956-5-19)
Supplement: Additional file 2 — Predicted transcription factors from the genes of the identified proteins in this study. [file 1477-5956-5-19-S2.pdf]

<table>
<tr><th>Spot number</th><th>Protein name</th><th>NCBI nr</th><th>SwissProt</th><th>Mr ob</th><th>pI ob</th><th>Location</th><th>PTS1/2</th><th>Cvage</th><th>Score</th><th>p</th><th>Source</th><th>Peroxisomal pathways</th></tr>
<tr><td>59,76-78,193,195</td><td>catalase</td><td>gi|6753272</td><td>P24270</td><td>60000</td><td>7.5-7.8</td><td>Px</td><td>ANL</td><td>30%</td><td>114</td><td>3.981E-07</td><td>1,2</td><td>auxiliary β-oxidation</td></tr>
<tr><td>11,100,145,161</td><td>acyl-CoA oxidase 1 (palmitoyl)</td><td>gi|66793429</td><td>Q9R0H0</td><td>25000;70000</td><td>8.7-9.0</td><td>Px</td><td>SKL</td><td>24%</td><td>60</td><td>0.1</td><td>1,2</td><td>straight-chain fatty acid β-oxi.</td></tr>
<tr><td>120,150</td><td>L- peroxisomal bifunctional enzyme (L-PBE)</td><td>gi|17366737</td><td>Q9DBM2</td><td>80000</td><td>9, 9.4</td><td>Px</td><td>SKL</td><td>28%</td><td>73</td><td>0.0050119</td><td>1,2</td><td>straight-chain fatty acid β-oxi.</td></tr>
<tr><td>51,52</td><td>3-ketoacyl-CoA thiolase A</td><td>gil30525893</td><td>Q921H8</td><td>40000</td><td>9.0</td><td>Px</td><td>PTS2</td><td>26%</td><td>66</td><td>0.0251189</td><td>1</td><td>straight-chain fatty acid β-oxi.</td></tr>
<tr><td>97,98,136,176,178</td><td>acetyl-Co A acyltransferase 1</td><td>gi|18700004</td><td>Q8LF48</td><td>43000</td><td>8.5, 9.0</td><td>Px</td><td>PTS2</td><td>20%</td><td>61</td><td>0.0794328</td><td>1,2</td><td>straight-chain fatty acid β-oxi.</td></tr>
<tr><td>179,197</td><td>isocitrate dehydrogenase</td><td>gi|1589591</td><td>P54071</td><td>50000</td><td>7.6,9.0</td><td>Px</td><td>PTS2</td><td>22%</td><td>59</td><td>0.1258925</td><td>2</td><td>straight-chain fatty acid β-oxi.</td></tr>
<tr><td>21-24,153,174,175</td><td>NADPH-dep retinol dehydrogenase/reductase</td><td>gi|32450664</td><td>Q99LB2</td><td>31000</td><td>8.9-9.3</td><td>Px</td><td>SRL</td><td>46%</td><td>84</td><td>0.0003981</td><td>1,2</td><td>straight-chain fatty acid β-oxi.</td></tr>
<tr><td>201</td><td>malonyl-CoA decarboxylase</td><td>gi|56797739</td><td>Q99J39</td><td>26000</td><td>7.4</td><td>Px</td><td>SKL</td><td>23%</td><td>57</td><td>0.1995262</td><td>2</td><td>straight-chain fatty acid β-oxi.</td></tr>
<tr><td>43</td><td>dienoyl-CoA isomerase</td><td>gi|2606086</td><td>O35459</td><td>37000</td><td>6.5</td><td>Px</td><td>SKL</td><td>21%</td><td>53</td><td>0.5011872</td><td>1</td><td>straight-chain fatty acid β-oxi.</td></tr>
<tr><td>141,194</td><td>D-peroxisomal bifunctional protein (D-PBP)</td><td>gi|31982273</td><td>P51660</td><td>34000;82000</td><td>7.2,8.8</td><td>Px</td><td>AKL</td><td>20%</td><td>58</td><td>0.1584893</td><td>2</td><td>branched-chain fatty acid</td></tr>
<tr><td>133-136,159</td><td>3-ketoacyl-CoA thiolase B</td><td>gi|22122797</td><td>Q8VCH0</td><td>45000;46000</td><td>9.6</td><td>Px</td><td>PTS2</td><td>40%</td><td>72</td><td>0.0063096</td><td>2</td><td>branched-chain fatty acid</td></tr>
<tr><td>7,147</td><td>sterol carrier protein 2</td><td>gi|26344327</td><td>P32020</td><td>15000</td><td>9.4</td><td>Px</td><td>AKL</td><td>46%</td><td>64</td><td>0.0398107</td><td>1,2</td><td>auxiliary β-oxidation</td></tr>
<tr><td>42</td><td>enoyl-CoA hydratase 1</td><td>gi|7949037</td><td>N/A</td><td>36000</td><td>7.0</td><td>Px</td><td>SKL</td><td>21%</td><td>53</td><td>0.5011872</td><td>1</td><td>auxiliary β-oxidation</td></tr>
<tr><td>66, 94, 95</td><td>sterol carrier protein 2</td><td>gi|45476581</td><td>P32020</td><td>55000; 60000</td><td>7, 7.6-7.8</td><td>Px</td><td>AKL</td><td>18%</td><td>76</td><td>0.0025119</td><td>1</td><td>auxiliary β-oxidation</td></tr>
<tr><td>170</td><td>peroxisomal 2-enoyl-CoA reductase</td><td>gi|13506791</td><td>Q99MZ7</td><td>32000</td><td>8.7</td><td>Px</td><td>AKL</td><td>30%</td><td>60</td><td>0.1</td><td>2</td><td>auxiliary β-oxidation</td></tr>
<tr><td>116,117</td><td>nudix</td><td>gi|25050637</td><td>P11930</td><td>42000</td><td>6.5</td><td>Px</td><td>AHL</td><td>19%</td><td>67</td><td>0.0199526</td><td>1</td><td>auxiliary β-oxidation</td></tr>
<tr><td>202-205</td><td>nudix</td><td>gi|14861848</td><td>P11930</td><td>41000</td><td>6.2-6.6</td><td>Px</td><td>AHL</td><td>56%</td><td>182</td><td>6.31E-14</td><td>3</td><td>auxiliary β-oxidation</td></tr>
<tr><td>41</td><td>phytanoyl-CoA hydroxylase</td><td>gi|6754564</td><td>O35386</td><td>36000</td><td>7.3</td><td>Px</td><td>PTS2</td><td>20%</td><td>64</td><td>0.0398107</td><td>1</td><td>α-oxidation</td></tr>
<tr><td>84</td><td>2-hydroxyphytanoyl-CoA lyase</td><td>gi|31560355</td><td>Q9QXE0</td><td>65000</td><td>6.8</td><td>Px</td><td>SNM</td><td>30%</td><td>104</td><td>3.981E-06</td><td>1</td><td>α-oxidation</td></tr>
<tr><td>50</td><td>α-methylacyl-CoA racemase</td><td>gil13626101</td><td>O09174</td><td>40000</td><td>7.7</td><td>Px</td><td>ANL</td><td>24%</td><td>70</td><td>0.01</td><td>1</td><td>α-oxidation</td></tr>
<tr><td>55</td><td>hydroxyacid oxidase 1/ glycolate oxidase 1</td><td>gi|6754156</td><td>Q9WU19</td><td>44000</td><td>8.7</td><td>Px</td><td>SKI</td><td>28%</td><td>61</td><td>0.0794328</td><td>1</td><td>α-oxidation</td></tr>
<tr><td>10</td><td>3-hydroxy-3-methylglutaryl-CoA synthase 2</td><td>gi|20965433</td><td>P54869</td><td>26000</td><td>8.7</td><td>Px/Mit</td><td>pigg-back</td><td>33%</td><td>68</td><td>0.0158489</td><td>1</td><td>isoprenoid biosynthesis</td></tr>
<tr><td>26</td><td>3-hydroxy-3-methylglutaryl CoA lyase</td><td>gi|409499</td><td>P38060</td><td>33000</td><td>9.1</td><td>Px/Mit</td><td>CKL</td><td>24%</td><td>70</td><td>0.01</td><td>1</td><td>isoprenoid biosynthesis related</td></tr>
<tr><td>38</td><td>D-amino acid oxidase 1</td><td>gil17390882</td><td>P18894</td><td>33000</td><td>6.0</td><td>Px</td><td>SHL</td><td>25%</td><td>65</td><td>0.0316228</td><td>1</td><td>amino acid metobolism</td></tr>
<tr><td>54</td><td>alanine-glyoxylate aminotransferase</td><td>gi|19388006</td><td>Q8R128</td><td>42000</td><td>7.8</td><td>Px</td><td>NKL</td><td>24%</td><td>63</td><td>0.0501187</td><td>1</td><td>amino acid metobolism</td></tr>
<tr><td>63</td><td>peroxisomal sarcosine oxidase</td><td>gi|20139923</td><td>Q9D826</td><td>47000</td><td>7.8</td><td>Px</td><td>AHL</td><td>21%</td><td>59</td><td>0.1258925</td><td>1</td><td>amino acid metobolism</td></tr>
<tr><td>85,86</td><td>epoxide hydrolase</td><td>gi|477004</td><td>P34914</td><td>65000</td><td>6.5-6.7</td><td>Px</td><td>SKI</td><td>32%</td><td>104</td><td>3.981E-06</td><td>1</td><td>amino acid metobolism</td></tr>
<tr><td>28-30,35,106-108,142,
152,165,167,187-190</td><td>urate oxidase</td><td>gi|18044669</td><td>P25688</td><td>35000</td><td>5.8-6.0,9.0-9.1</td><td>Px</td><td>SRL</td><td>26%</td><td>63</td><td>0.0501187</td><td>1,2</td><td>purine/pirimidine metabolism</td></tr>
<tr><td>49</td><td>similar to ornithine transcarbamylase</td><td>gi|19353187</td><td>P11725</td><td>40000</td><td>7.6</td><td>Px pred</td><td>PTS2-like</td><td>15%</td><td>57</td><td>0.1995262</td><td>1</td><td>purine/pirimidine metabolism</td></tr>
<tr><td>83</td><td>ornithine aminotransferase</td><td>gi|8393866</td><td>P29758</td><td>49000</td><td>5.8</td><td>Px pred</td><td>PTS2</td><td>25%</td><td>109</td><td>1.259E-06</td><td>1</td><td>purine/pirimidine metabolism</td></tr>
<tr><td>32</td><td>peroxisome assembly factor-1</td><td>gi|450862</td><td>P55098</td><td>34000</td><td>6.8</td><td>Px</td><td>NAL</td><td>23%</td><td>58</td><td>0.1584893</td><td>1</td><td>peroxisome biogenesis</td></tr>
<tr><td>87</td><td>leukotriene A4 hydrolase</td><td>gi|6678726</td><td>P24527</td><td>67000</td><td>6.1</td><td>Px/Cyt</td><td>PTS2-like</td><td>18%</td><td>60</td><td>0.1</td><td>1</td><td>putative peroxisomal protein</td></tr>
<tr><td>101</td><td>glutathione S-transferase</td><td>gi|10092608</td><td>P19157</td><td>29000</td><td>8.1</td><td>Px pred</td><td>PTS2-like</td><td>22%</td><td>64</td><td>0.0398107</td><td>1</td><td>putative peroxisomal protein</td></tr>
<tr><td>33</td><td>lactamase</td><td>gi|13278495</td><td>N/A</td><td>34000</td><td>6.7</td><td>Px/Mit</td><td>AVL</td><td>26%</td><td>63</td><td>0.0501187</td><td>1</td><td>putative peroxisomal protein</td></tr>
<tr><td>88,93</td><td>60 kDa heat shock protein</td><td>gi|76779273</td><td>P63038</td><td>62000</td><td>5.7-5.9</td><td>Px/Mit</td><td>PTS2</td><td>21%</td><td>59</td><td>0.1258925</td><td>1</td><td>putative peroxisomal protein</td></tr>
<tr><td>8</td><td>Cu-Zn superoxide dismutase</td><td>gil309207</td><td>P08228</td><td>20000</td><td>6.8</td><td>Px/Cyt</td><td></td><td>23%</td><td>65</td><td>0.0316228</td><td>1</td><td>putative peroxisomal protein</td></tr>
<tr><td>60</td><td>similar to DOCK protein</td><td>gi|26329657</td><td>Q8BUR4</td><td>45000</td><td>7.1</td><td>Px/Cyt</td><td>GNL</td><td>21%</td><td>56</td><td>0.2511886</td><td>1</td><td>putative peroxisomal protein</td></tr>
<tr><td>27,104</td><td>carbonic anhydrase III</td><td>gi|226778</td><td>P16015</td><td>33000</td><td>7.5</td><td>Px/Cyt</td><td>SKF</td><td>24%</td><td>67</td><td>0.0199526</td><td>1</td><td>putative peroxisomal protein</td></tr>
<tr><td>109</td><td>70kDa heat shock protein</td><td>gi|2506545</td><td>N/A</td><td>75000</td><td>4.6</td><td>Px/ER</td><td></td><td>26%</td><td>78</td><td>0.0015849</td><td>1</td><td>putative peroxisomal protein</td></tr>
<tr><td>155</td><td>unnamed protein product</td><td>gi|26383558</td><td>N/A</td><td>50000</td><td>7.6</td><td>Px/Nc</td><td></td><td>29%</td><td>57</td><td>0.1995262</td><td>2</td><td>putative peroxisomal protein</td></tr>
<tr><td>73, 74, 79, 69</td><td>aldehyde dehydrogenase 2</td><td>gi|6753036</td><td>P47738</td><td>55-60000</td><td>6.6-7.1</td><td>Px/Mit/Cyt</td><td>PTS2-like</td><td>40%</td><td>117</td><td>1.995E-07</td><td>1</td><td>putative peroxisomal protein</td></tr>
<tr><td>6</td><td>fatty acid binding protein</td><td>gi|8393343</td><td>P12710</td><td>16000</td><td>9.0</td><td>Cyt</td><td></td><td>24%</td><td>58</td><td>0.1584893</td><td>1</td><td></td></tr>
<tr><td>16</td><td>short chain L-3-hydroxyacyl-CoA dehydrogenase</td><td>gi|13182962</td><td>Q99N15</td><td>30000</td><td>9.0</td><td>Cyt</td><td></td><td>39%</td><td>60</td><td>0.1</td><td>1</td><td></td></tr>
<tr><td>37</td><td>betaine-homocysteine methyltransferase</td><td>gi|7709990</td><td>O35490</td><td>34000</td><td>7.8</td><td>Cyt</td><td></td><td>28%</td><td>63</td><td>0.0501187</td><td>1</td><td></td></tr>
<tr><td>47</td><td>protein tyrosine phosphatase</td><td>gi|7305423</td><td>P28563</td><td>38000</td><td>7.5</td><td>Cyt</td><td></td><td>20%</td><td>59</td><td>0.1258925</td><td>1</td><td></td></tr>
<tr><td>56</td><td>glutamine synthetase</td><td>gi|15419027</td><td>P15105</td><td>42000</td><td>7.5</td><td>Cyt</td><td></td><td>28%</td><td>59</td><td>0.1258925</td><td>1</td><td></td></tr>
<tr><td>140,156</td><td>aldolase 2</td><td>gi|21707669</td><td>Q91Y97</td><td>40000</td><td>8.7,9.0</td><td>Cyt</td><td></td><td>36%</td><td>67</td><td>0.0199526</td><td>1</td><td></td></tr>
<tr><td>31,160</td><td>short chain 3-hydroxyacyl-CoA dehydrogenase</td><td>gi|21431780</td><td>Q61425</td><td>30000;33000</td><td>8.8</td><td>Mit</td><td></td><td>24%</td><td>55</td><td>0.3162278</td><td>1,2</td><td></td></tr>
<tr><td>34</td><td>3-mercaptopyruvate sulfurtransferase</td><td>gi|65301475</td><td>Q99J99</td><td>33000</td><td>7.8</td><td>Mit</td><td></td><td>28%</td><td>77</td><td>0.0019953</td><td>1</td><td></td></tr>
<tr><td>163,164</td><td>thiosulfate sulfurtransferase</td><td>gi|6678449</td><td>P52196</td><td>38000</td><td>7.5</td><td>Mit</td><td></td><td>35%</td><td>58</td><td>0.1584893</td><td>2</td><td></td></tr>
<tr><td>44</td><td>pyruvate dehydrogenase</td><td>gi|18043470</td><td>Q9D051</td><td>37000</td><td>6.1</td><td>Mit</td><td></td><td>24%</td><td>54</td><td>0.3981072</td><td>1</td><td></td></tr>
<tr><td>46,169,177</td><td>malate dehydrogenase</td><td>gi|6678916</td><td>P08249</td><td>38000</td><td>8.6</td><td>Mit</td><td></td><td>28%</td><td>58</td><td>0.1584893</td><td>1,2</td><td></td></tr>
<tr><td>53,64,65</td><td>similar to acetyl-CoA acyltransferase 2</td><td>gi|20810027</td><td>Q8BWT1</td><td>44000; 46000</td><td>7.8-9-0</td><td>Mit</td><td></td><td>23%</td><td>70</td><td>0.01</td><td>1</td><td></td></tr>
<tr><td>124-127</td><td>acetyl CoA acyltransferase 2</td><td>gi|29126205</td><td>Q99JY0</td><td>50000;52000</td><td>8.0-8.4</td><td>Mit</td><td></td><td>38%</td><td>73</td><td>0.0050119</td><td>2</td><td></td></tr>
<tr><td>58</td><td>acetyl-CoA dehydrogenase</td><td>gi|16740777</td><td>N/A</td><td>44000</td><td>7.5</td><td>Mit</td><td></td><td>26%</td><td>68</td><td>0.0158489</td><td>1</td><td></td></tr>
<tr><td>89,90</td><td>peptide-binding protein 74</td><td>gi|14917005</td><td>P38647</td><td>75000</td><td>6.0-6.2</td><td>Mit</td><td></td><td>25%</td><td>63</td><td>0.0501187</td><td>1</td><td></td></tr>
<tr><td>62,137-139</td><td>glutamte oxalacetate transaminase</td><td>gil6754036</td><td>P05202</td><td>44000</td><td>9.2</td><td>Mit</td><td></td><td>21%</td><td>93</td><td>5.012E-05</td><td>1,2</td><td></td></tr>
<tr><td>67,71,72</td><td>glutamate dehydrogenase</td><td>gi|6680027</td><td>P26443</td><td>53000;60000</td><td>7.4-7.5</td><td>Mit</td><td></td><td>22%</td><td>64</td><td>0.0398107</td><td>1</td><td></td></tr>
<tr><td>92</td><td>sarcosine dehydrogenase</td><td>gi|20149748</td><td>Q99LB7</td><td>95000</td><td>7</td><td>Mit/Cyt</td><td></td><td>30%</td><td>78</td><td>0.0015849</td><td>1</td><td></td></tr>
<tr><td>57,118,119</td><td>similar to carbamoyl-phosphate synthase</td><td>gi|82879179</td><td>Q8C196</td><td>150000</td><td>6.8-7.2</td><td>Mit</td><td></td><td>31%</td><td>107</td><td>1.995E-06</td><td>1</td><td></td></tr>
<tr><td>48, 131, 132</td><td>trifunctional enzyme</td><td>gi|26345684</td><td>Q99JY0</td><td>38000;52000</td><td>6.5,9.3-9.5</td><td>Mit</td><td></td><td>25%</td><td>67</td><td>0.0199526</td><td>1,2</td><td></td></tr>
<tr><td>154</td><td>serine hydroxymethyl transferase 2</td><td>gi|21312298</td><td>Q9SZJ5</td><td>58000</td><td>8.2</td><td>Mit</td><td></td><td>26%</td><td>59</td><td>0.1258925</td><td>2</td><td></td></tr>
<tr><td>171</td><td>D-β-hydroxybutyrate dehydrogenase</td><td>gi|31982169</td><td>Q80XN0</td><td>31000</td><td>9</td><td>Mit</td><td></td><td>26%</td><td>55</td><td>0.3162278</td><td>2</td><td></td></tr>
<tr><td>143</td><td>enoyl CoA hydratase 1</td><td>gi|29789289</td><td>Q8BH95</td><td>31000</td><td>7.5</td><td>Mit</td><td></td><td>24%</td><td>59</td><td>0.1258925</td><td>2</td><td></td></tr>
<tr><td>81,111</td><td>protein disulfide isomerase</td><td>gi|129729</td><td>P09103</td><td>59-61000</td><td>5.0,6.5</td><td>ER</td><td></td><td>28%</td><td>64</td><td>0.0398107</td><td>1</td><td></td></tr>
</table>
